# Effects of long-term antipsychotics treatment on body weight: A population-based cohort study

**DOI:** 10.1177/0269881119885918

**Published:** 2019-11-14

**Authors:** Juan Carlos Bazo-Alvarez, Tim P Morris, James R Carpenter, Joseph F Hayes, Irene Petersen

**Affiliations:** 1 Research Department of Primary Care and Population Health, University College London, London, UK; 2 Instituto de Investigación, Universidad Católica los Ángeles de Chimbote, Peru; 3 MRC Clinical Trials Unit at University College London, London, UK; 4 Department of Medical Statistics, London School of Hygiene and Tropical Medicine, London, UK; 5 Division of Psychiatry, University College London, London, UK; 6 Department of Clinical Epidemiology, Aarhus University, Denmark

**Keywords:** Antipsychotic agents, dopamine, serotonin, noradrenaline, weight gain, electronic health records, interrupted time series analysis

## Abstract

**Background:**

Antipsychotics are often prescribed for long-term periods, however, most evidence of their impact on body weight comes from short-term clinical trials. Particularly, impact associated with dosage has been barely studied.

**Aims:**

The aim of this study was to describe the short- and long-term change in body weight of people initiated on high or low doses of the three most commonly prescribed second-generation antipsychotics.

**Methods:**

Retrospective cohorts of individuals with a diagnosed psychotic disorder observed from 2005 to 2015 in the UK primary care. The exposure was the first prescription of olanzapine, quetiapine or risperidone. The main outcome was change in body weight four years before and four years after initiation of antipsychotic treatment, stratified on sex and ‘low’ or ‘high’ dose.

**Results:**

In total, 22,306 women and 16,559 men were observed. Olanzapine treatment was associated with the highest change in weight, with higher doses resulting in more weight gain. After 4 years, given a high dose of olanzapine (> 5 mg), women gained on average +6.1 kg; whereas given a low dose (⩽ 5 mg), they gained +4.4 kg. During the first six weeks of olanzapine treatment, they gained on average +3.2 kg on high dose and +1.9 kg on low dose. The trends were similar for men. Individuals prescribed risperidone and quetiapine experienced less weight gain in both the short- and long-term.

**Conclusions:**

Olanzapine treatment was associated with the highest increase in weight. Higher doses were associated with more weight gain. Doctors should prescribe the lowest effective dose to balance mental-health benefits, weight gain and other adverse effects.

## Introduction

Overweight and obesity is a worldwide problem that impacts severely on population health (Newcomer and Haupt, 2006). Since the prevalence of overweight and obesity is higher in individuals with severe mental illnesses than in the general population (Elmslie et al., 2000; Holt and Peveler, 2009), their risk of harmful consequences is also higher (Hayes et al., 2017; Osborn et al., 2007). Individuals with severe mental illnesses are more susceptible to developing metabolic syndrome, type-2 diabetes mellitus (De Hert et al., 2006) and cardiovascular diseases (Emul and Kalelioglu, 2015; Osborn et al., 2007), leading to a higher risk of death. Adults with schizophrenia have three and a half times the mortality risk than the general population, with cardiovascular diseases the most common cause (Olfson et al., 2015; Osborn et al., 2007). Particularly, Lahti et al. demonstrated that the risk of death is higher in women than in men with schizophrenia (Lahti et al., 2012), suggesting that differences between sexes need to be further investigated.

Second-generation antipsychotics (AP) are a known cause of weight gain (Bak et al., 2014; Osborn et al., 2018). Some evidence suggests that women gain more weight than men during AP treatment (Seeman, 2008). One study suggested that women have five times the odds of increasing body mass index (BMI) compared with men after a period of two years or more (Koga, 2003). Of women treated with clozapine, 29% gained ⩾ 20% of their baseline body weight after two years of follow up, in contrast to 13% of men (Covell et al., 2004). Other studies have demonstrated similar differences between men and women (Gebhardt et al., 2009; Najar et al., 2017). However, most of these studies are based on small sample sizes of less than 200 individuals, and most do not distinguish between short- and long-term weight gain associated with antipsychotic treatment.

Weight gain after initiation of antipsychotic treatment may also depend on body weight when treatment is initiated. Thus, Gebhardt et al. found that low BMI before first AP treatment predicted a faster increment of BMI after treatment initiation (Gebhardt et al., 2009), and a similar conclusion was reached by Najar et al. (2017). On the other hand, we have limited information about how doses of antipsychotic treatment are associated with weight gain (Bak et al., 2014).

In this study, our aim was to investigate the change in body weight of patients initiated with high or low doses of the three most commonly prescribed second-generation antipsychotics: olanzapine, risperidone and quetiapine. Our objectives were to evaluate:

(1) the short- and long-term change in body weight in men and women upon initiation of AP;

(2)  whether this is different for low and high doses;

(3) whether low body weight at treatment initiation had the greatest weight gain.

## Methods

### Data source

We used anonymized, longitudinal patients’ records from The Health Improvement Network (THIN), a database that comprises information from UK primary care electronic health records from general practices (Roland et al., 2012). THIN integrates more than 12 million patients from 711 general practices, including demographic data (sex, year of birth and indicator of social deprivation (quintiles of Townsend score)) and clinical data. The clinical data are recorded using the hierarchical Read Code system (Chisholm, 1990). In the UK, more than 95% are registered with a general practice, THIN is roughly representative of the UK population and has been previously used for reporting health indicators at the national level (Blak et al., 2011). For this study, we included data from all practices after they have been deemed to be operating at the standard of acceptable computer usage (Horsfall et al., 2013) and whose reported mortality rate was consistent with national statistics (Maguire et al., 2009).

### Study population

At the individual level, we included all patients aged between 18 and 99 years at the date they started their first treatment with olanzapine, risperidone or quetiapine; between 1 January 2005 and 31 December 2015. We included patients with a diagnosed psychiatric disorder (schizophrenia, bipolar disorder, other non-affective psychoses, borderline personality disorder, anxiety, depression or dementia) who had at least one further prescription of the same AP within three months after the first prescription. We judged that these individuals were more likely to have initiated treatment than those with a single prescription. Patients who had been initiated on more than one type of AP were excluded (including switchers). A few individuals had no records of year of birth, sex or social deprivation records and were thus excluded from our study. Likewise, we excluded individuals with no available data 12 months before the date of initiation of antipsychotic treatments since they may have initiated antipsychotic treatment elsewhere.

### Variables and measurements

The exposure of interest was the initiation of olanzapine, risperidone or quetiapine prescription. In the Neuroscience-based Nomenclature olanzapine is a dopamine and serotonin receptor antagonist, risperidone is a dopamine, serotonin and norepinephrine receptor antagonist, and quetiapine is a dopamine and serotonin receptor antagonist and norepinephrine reuptake inhibitor (Nutt and Blier, 2016). The outcome was body weight, measured in kilograms. The main covariates were sex (women/men) and first prescribed dose of AP (hereafter called ‘first dose’). All AP reported first doses in milligrams, but we used the dose-equivalence approach of Woods (Woods, 2003) for defining cut-off points of low/high first dose: ⩽ 5 mg for olanzapine, ⩽ 75 mg for quetiapine and ⩽ 2 mg for risperidone. Using the ‘2 mg of haloperidol equals 100 mg of chlorpromazine’ convention as reference, Woods (2003) explored available evidence for identifying the minimum effective dose across olanzapine, quetiapine and risperidone, defining this dose equivalence. The first dose is a good predictor of all subsequent doses prescribed during treatment; thus, over time, patients usually stay in a dose range close to the first dose they were prescribed (data not shown). We also retrieved information on age, height, social deprivation (Townsend score 1–5, from least to most deprived), smoking and drinking status, having a type-2 diabetes mellitus diagnosis, systolic blood pressure (SBP), low-density lipoprotein cholesterol (LDL-cholesterol) and high-density lipoprotein cholesterol (HDL-cholesterol); recorded within first year of initiation of treatment. This information served mostly for sample characterization; only sex, age, type-2 diabetes mellitus diagnosis and social deprivation were fully observed.

### Statistical analysis

We used an interrupted time series approach (Bernal et al., 2017) to analyse weight change over time, with one model for each of the three AP initiation cohorts by sex (six models in total, one per drug per sex). We modelled weight change over time using continuous linear splines with random intercept and slopes models (unstructured covariance, restricted maximum likelihood), from which three slopes of weight change were estimated for: (a) –4 years to baseline (pre-treatment), (b) baseline to +6 weeks (short-term), (c) +6 weeks to +4 years (long-term). Differences between slopes served to describe weight change after AP treatment initiation, both crude and adjusted for age and social deprivation (objective 1). The correlation between average weight at baseline (intercept) and short-term gradient of change (short-term slope) was estimated, as it provided an estimate for whether individuals with lower weight at baseline gain more or less weight after AP treatment initiation than individuals with higher body weight. Negative correlations mean that individuals with low weight gain more weight during the short-term period and vice versa (objective 3). Our main analysis was performed after stratifying each of cohorts according to low/high first dose. This was to examine whether the gradient of weight change after treatment varies between low/high first doses of AP (objective 2). For all these models, the Intraclass Correlation Coefficient (ICC) was reported. Likelihood ratio tests were performed to compare the goodness of fit between the nested models. We assumed weight records were missing at random within strata, conditional on observed weights, so that modelling the observed data over time provides unbiased estimates (van Buuren, 2012). We also assumed missing data on dose was missing at random, so that the complete case analysis we performed provides unbiased estimates (White and Carlin, 2010). Model assessment included evaluation of residuals and a visual exploration of average and individual trajectories. Although the chosen impact model (linear splines with knots at baseline and +6 weeks) was informed by both the clinical criteria and evidence (Bak et al., 2014), we also performed a sensitivity analysis following the suggestions from Lopez Bernal et al. (Bernal et al., 2017). This sensitivity analysis consisted of comparing our preferred linear spline model against another feasible impact model, a restricted cubic spline model (knots again at baseline and +6 weeks), using graphical and analytical tools (see Figure S1 in supplemental material). Estimates are given with 95% confidence intervals (CIs). All the statistical analyses were performed using Stata 15 for Windows (Stata, 2017).

## Results

**Figure 1.**
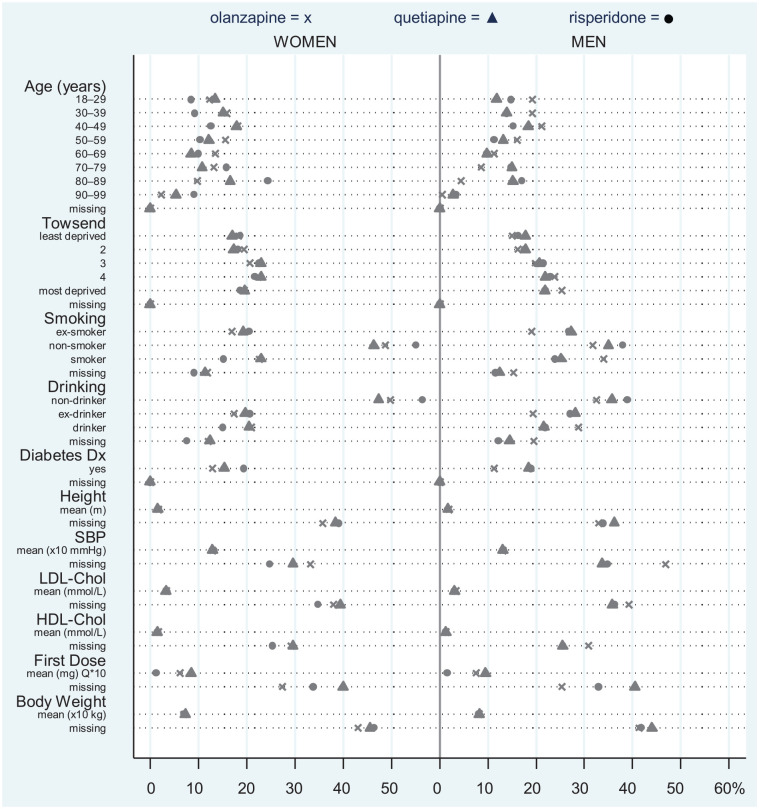
Baseline characteristics of patients from olanzapine, quetiapine and risperidone cohorts, stratified by sex. From height onwards, some continuous variables changed their scale as labelled below their names.

In total, we included 16,559 men and 22,306 women in the study. The median number ± interquartile range of weight measurements within individual trajectories over eight years of observation were 6 ± 7 and 8 ± 10 (olanzapine cohorts), and 7 ± 8 and 8 ± 9 (quetiapine and risperidone cohorts) for men and women respectively. Characteristics of the individuals are summarized in Figure 1 and are provided in more detail in Table S1. On average, at initiation of treatment, men were younger than women prescribed olanzapine (men = 47.5 years ±17.8 SD, women = 54.0 years ±19.5 SD) and risperidone (men = 56.6 years ±22.1 SD, women = 64.5 years ±21.8 SD), but were of similar age in the quetiapine cohort (men = 56.5 years ±20.7 SD, women = 56.1 years ±22.1 SD). On average, men were prescribed higher dose of olanzapine (+1 mg), quetiapine (+10 mg) and risperidone (+0.3 mg).

**Figure 2.**
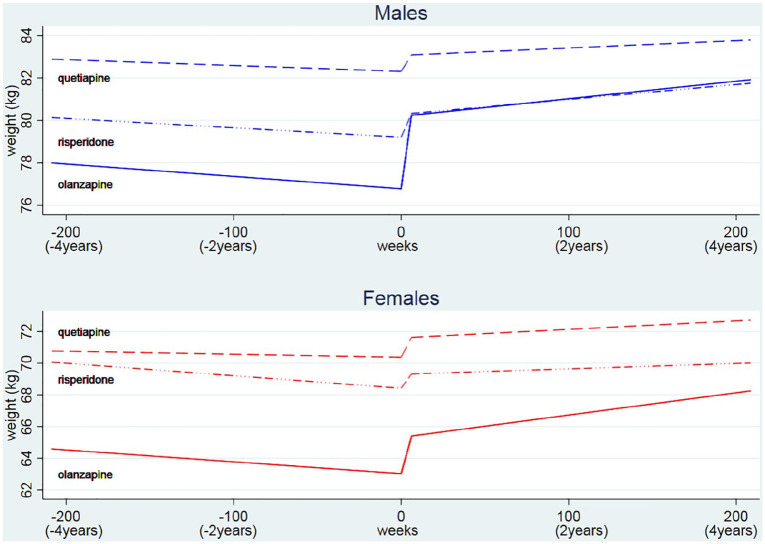
Changes in body weight over time before and after treatment initiation by drugs and sex.

In the short (< 6 weeks) and long term (⩾ 6 weeks to ⩽ 4 years), individuals treated with any of the three AP drugs gained weight, especially those patients prescribed olanzapine. Pre-treatment weight change was negligible for quetiapine (women and men) and risperidone (men only) cohorts, and slightly negative for the rest of cohorts. In the short-term after olanzapine initiation, men’s weight increased by 0.569 kg/week (3.4 kg over the first six weeks) and women’s weight increased by 0.382 kg/week (2.3 kg over the first six weeks) (Tables 1 and S2). Individuals initiated on quetiapine and risperidone also gained weight shortly after initiation of treatment, but to a lesser extent (Tables 1 and S2, and Figure 2). Individuals continued to gain weight after six weeks, but at a slower rate than the first six weeks. For example, for women initiated on olanzapine, long-term weight gain was estimated to be 0.014 kg/week (0.7 kg per year) (Tables 1 and S2, and Figure 2). Women who were initiated on olanzapine were in general slightly lighter (69.7 kg) than women initiated on risperidone (73.3 kg) and quetiapine (70.1 kg), but there was not much difference for the men (weight at baseline, see Figure 1 and Table S2). Women who had a lower weight before initiation of olanzapine gained more weight in the short term than women who had a higher weight (correlation between intercept and slope = –0.068, 95% CI: –0.121 to −0.014); a similar effect was observed for men (correlation between intercept and slope = –0.050, 95% CI: –0.113 to +0.014) (Table S2).

**Table 1.** Expected weight gain for an average patient prescribed a particular antipsychotic, stratified by dose and sex.

Drug	Sex	*n* ^a^	Dose^b^	Weight gained during **short-time** (0–6 weeks), kg	95% CI	Weight gained during **long-time** (6 weeks–4 years), kg	95% CI	Total weight gained
OLANZAPINE (*n* = 9499)	Women	5004	Overall	2.3	(1.9–2.7)	2.8	(2.2–3.5)	5.1
		*2535*	Low	1.9	(1.4–2.4)	2.5	(1.6–3.3)	4.4
		*1100*	High	3.2	(2.4–4.0)	2.9	(1.6–4.2)	6.1
	Men	4495	Overall	3.4	(3.0–3.8)	1.7	(0.9–2.4)	5.1
		*1887*	Low	2.6	(2.0–3.2)	1.9	(0.8–3.0)	4.5
		*1470*	High	4.5	(3.6–5.3)	1.4	(0.2–2.7)	5.9
QUETIAPINE (*n* = 19,965)	Women	12,149	Overall	1.2	(1.0–1.5)	1.1	(0.6–1.6)	2.3
		*5372*	Low	0.7	(0.3–1.0)	0.9	(0.1–1.6)	1.6
		*1912*	High	2.3	(1.6–2.9)	1.6	(0.4–2.7)	3.9
	Men	7816	Overall	0.8	(0.4–1.1)	0.7	(0.1–1.3)	1.5
		*3326*	Low	0.5	(0.0–0.9)	−0.7	(–1.8–0.3)	−0.3
		*1326*	High	1.6	(0.9–2.4)	1.0	(–0.3–2.2)	2.6
RISPERIDONE (*n* = 9401)	Women	5153	Overall	0.9	(0.5–1.3)	0.7	(–0.1–1.5)	1.6
		*3102*	Low	1.0	(0.5–1.4)	0.1	(–0.9–1.1)	1.1
		*316*	High	1.1	(–0.7–2.9)	3.5	(1.0–5.9)	4.6
	Men	4248	Overall	1.1	(0.6–1.5)	1.4	(0.4–2.4)	2.5
		*2411*	Low	1.0	(0.4–1.7)	1.1	(–0.3–2.6)	2.2
		*441*	High	1.9	(0.5–3.3)	1.4	(–0.7–3.5)	3.3

a Overall estimates come from Table S2 (*n* = 38,865) and low/high dose estimates come from Table S3 (*n* = 25,198). *n* from Table S2 < *n* from Table S3 due to missing data on dose.

b Cut off point for low/high dose was: ⩽ 5 mg for Olanzapine, ⩽ 75 mg for Quetiapine and ⩽ 2 mg for Risperidone.

The weight gain in individuals who were initiated on high dose of AP was greater than those initiated on low dose. When olanzapine was initiated at high dose (> 5 mg), women gained +0.534 kg/week (+3.2 kg over 6 weeks) and men +0.743 kg/week (+4.5 kg over 6 weeks) compared with low-dose gain of +0.314 kg/week (+1.9 kg over 6 weeks) for women and +0.425 kg/week (+2.6 kg over 6 weeks) for men (Tables 1 and S3). The short-term effect of initiation of quetiapine was also stronger for those given high doses (> 75 mg) (women +2.3 kg and men +1.6 kg, both over 6 weeks) than given low doses (women +0.7 kg and men +0.5 kg, both over 6 weeks). However, there was a relatively small difference for those initiated on risperidone low doses (⩽ 2 mg) (+1.0 kg over 6 weeks for both women and men) and high doses (women +1.1 kg and men 1.9 kg, both over 6 weeks). In the short-term, those given low doses of olanzapine tended to gain more weight as their weight at baseline was lower (women: correlation between intercept and slope = –0.155, 95% CI: –0.230 to −0.078; men: correlation between intercept and slope = –0.135, 95% CI: –0.235 to −0.033).

Cumulative weight gain in the long-term was particularly high in patients prescribed olanzapine but, for any drug, people did not on average lose the extra-weight they gained during the short-term (Table 1 and S3). For example, after four years from the first olanzapine prescription, a typical woman gained 2.3 kg (short-term, 95% CI: 1.9–2.7 kg) + 2.8 kg (long-term, 95% CI: 2.2–3.5 kg)  = 5.1 kg (total) whereas a typical man gained 3.4 kg (short-term, 95%CI: 3.0–3.8 kg) + 1.7 kg (long-term, 95% CI: 0.9–2.4 kg) = 5.1 kg (total) of AP induced extra-weight. The prescribed dose of olanzapine was also critical, particularly for women in the long-term. For example, given a low dose (< 5 mg), women gained 1.9 + 2.5 = 4.4 kg after four years; given a high dose (> 5 mg), women gained 3.2 + 2.9 = 6.1 kg. A similar impact of higher doses was observed for quetiapine and risperidone (Table 1 and S3).

## Discussion

This retrospective cohort study reports data from patients seen in primary care, before and after AP treatment initiation. Pre-treatment weight change was insignificant or slightly negative for all cohorts during four years before baseline. Individuals starting treatment with any AP gained weight on average, especially those patients prescribed olanzapine. Weight gain was much more rapid in the short-term than in the long-term. People who were initiated on high-dose AP experienced much greater absolute weight gain than those initiated on low dose AP. Cumulative weight gain during the long-term was particularly high in individuals treated with olanzapine but, for all APs, people typically never lost the extra weight they gained during the first six weeks of AP treatment.

### Strengths and limitations of this study

This study presents evidence from a large sample (*n* > 38,000) of people prescribed antipsychotic medications, taken from a population which is broadly representative of the UK (Blak et al., 2011). Patients prescribed antipsychotics are often treated for long periods, and so quantifying the risk of long-term side effects is particularly important. Clinical trials invariably fail to do this because of their short durations and much smaller sample size, so our study provides a necessary long-term perspective. We applied an analysis approach that has not been used previously in assessing AP-induced weight gain. A major advantage of our approach is that it includes pre-treatment weight change information, so patients act as their own controls in the analysis and any additional weight change after baseline is attributable to the AP treatment. The approach utilizes all individual weight records at their time of measurement, therefore avoiding the loss of information seen in previous studies which categorize outcomes or use period means or incidence rates as summary measures (Bak et al., 2014; Osborn et al., 2018). Our longitudinal model-based approach also accounts for missing weight records – assuming weight recording is missing at random within strata, conditional on observed weight measurements (Haneuse et al., 2016) – while incorporating informative pre-baseline weight data. From this method we expect unbiased estimates if data were missing at random (Molenberghs et al., 2014), a property that is not ensured by complete case analyses applied elsewhere (Bushe et al., 2012; Osborn et al., 2018). Following standard recommendations (Bernal et al., 2017), we guaranteed good statistical power by having equal periods of observation before and after baseline, and a large sample size. Additional analyses showed that our proposed linear spline models were very similar to the restricted cubic splines models (Figure S1), and for primary analysis we used the former as interpretation is more straightforward.

Our study does have a number of potential limitations. Information on possible time-varying confounders (for example, symptoms level or illness severity) was not included, however, it is reasonable to assume limited variation from patients’ baseline values for unmeasured confounders. Treatment initiation has been defined using first prescription date in general practice; but, for some individuals, the first prescription date might occur while the individual is under the care of secondary care mental services (these data are not recorded in primary care). However, it is most likely these patients have a first prescription date very close to the one in primary care, thus no major impact on estimates is expected.

We did not control for drugs prescribed to reduce antipsychotic-induced weight gain, or for multiple prescriptions of other drugs that could potentially affect weight as well. However, we know that drugs prescribed for ameliorating weight gain would only reduce the estimate of the real weight gain of the target population, thus we are not overestimating the weight gain effect. We did not assess weight gain associated with other antipsychotic medications as there were not enough data on them, but the three drugs included in this study are the most commonly prescribed antipsychotic medications in the UK (Marston et al., 2014) and have previously been associated with weight gain (Osborn et al., 2018). The weight gain trajectories we described are averages, thus they should be interpreted as typical patient trajectories. In practice, individual patients’ weight gain will vary from these average trajectories. However, the first weeks of treatment are critical for everyone. Finally, we did not control the number of prescriptions beyond the second prescription (treatment duration), meaning that studied patients can include those treated for long periods, those treated sporadically, just for a short period, or those who did not adhere to treatment regularly. This lack of control may reduce our long-term estimates of weight gain, but, given the evidence about dosage, we anticipate that patients exposed to AP on a regular basis and for long periods will have larger estimates of long-term weight gain.

### Comparison with other studies

Previous studies have suggested olanzapine is associated with a large short-term weight gain whereas risperidone and quetiapine have a moderate effect on weight (Bak et al., 2014). In the long-term, contrary to one previous finding (Haddad, 2005), we found that weight gain did not stabilize during four years of follow up. However, our finding of long-term effect of weight gain is consistent with previous studies by Bushe et al. (2012) and Osborn et al. (2018), but we are able to quantify the effect more accurately. Previous research has suggested women’s weight is more affected by AP exposure (Seeman, 2008); however, we found that only olanzapine (in the long-term) and quetiapine (in the long and short-term) induced more weight gain in women. Since our study population is a mixture of naïve and recurrent antipsychotic consumers, short- and long-term weight gain in olanzapine naïve individuals and long-term weight gain in risperidone naïve individuals can be higher than the weight gain reported by us (Bak et al., 2014). Risperidone seemed to be associated with greater weight gain in men than women both in the short- and long-term, and men prescribed olanzapine gained more weight in the short-term. Regarding the dosage, one recent study reanalysed results of 14 clinical trials to explore variations in weight gain across doses of olanzapine and risperidone (Spertus et al., 2018). Their conclusions about olanzapine are consistent with our results; that the excess risk of at least 7% weight gain is 16.1% for low doses (0–10 g chlorpromazine equivalent dose) and 46.8% for high doses (0–20 g chlorpromazine equivalent dose). They could not be conclusive about the effects of risperidone as they showed only a trend in weight gain; however, this trend is in line with our findings. Some advantages from our original study are: (a) we added similar information about quetiapine, (b) we observed longer periods of weight change (four years) and (c) we analysed information at individual-level from cohorts with more than 38,000 patients in total.

### Conclusions and policy implications

Over a four-year period, olanzapine treatment was associated with the highest increase in weight with around 6 kg for those on high dose and 4.5 kg for those on low dose. The weight gain was less dramatic for individuals treated with quetiapine and risperidone. In general, individuals did not lose the weight gained during the first six weeks of treatment. Doctors and patients may want to take the issue of a substantial weight gain into consideration when making decisions on initiation of antipsychotic treatments, and doctors should prescribe the lowest effective dose to balance mental health benefits, weight gain and other adverse effects.

## Supplemental Material

190503_Supplemental_Material – Supplemental material for Effects of long-term antipsychotics treatment on body weight: A population-based cohort study Supplemental material, 190503_Supplemental_Material for Effects of long-term antipsychotics treatment on body weight: A population-based cohort study by Juan Carlos Bazo-Alvarez, Tim P Morris, James R Carpenter, Joseph F Hayes and Irene Petersen in Journal of Psychopharmacology
