# Supplementary material for: Effects of long-term antipsychotics treatment on body weight: A population-based cohort study
Source: J Psychopharmacol. 2019 Nov 14;34(1):79–85. doi: 10.1177/0269881119885918 (PMC6947810; doi:10.1177/0269881119885918)
Supplement: 190503_Supplemental_Material – Supplemental material for Effects of long-term antipsychotics treatment on body weight: A population-based cohort study [file 190503_Supplemental_Material.docx]

# Supplemental Material

| *Table S1. Baseline characteristics of patients using Olanzapine, Quetiapine or Risperidone (3 retrospective cohorts), by sex.* | | | | | | | | | | | | | | | | | | | | | | | | | |
| --- | --- | --- | --- | --- | --- | --- | --- | --- | --- | --- | --- | --- | --- | --- | --- | --- | --- | --- | --- | --- | --- | --- | --- | --- | --- |
|  | | *OLANZAPINE (N=9499)* | | | | | | | | *QUETIAPINE (N=19965)* | | | | | | | | *RISPERIDONE (N=9401)* | | | | | | | |
|  |  | *Women* | | | | *Men* | | | | *Women* | | | | *Men* | | | | *Women* | | | | *Men* | | | |
|  |  | *N=5004 (52.7%)* | | | | *N=4495 (47.3%)* | | |  | *N=12149 (60.9%)* | | | | *N=7816 (39.1%)* | | |  | *N=5153 (54.8%)* | | | | *N=4248 (45.2%)* | | |  |
|  |  | **n** | **(** | **%** | **)** | **n** | **(** | **%** | **)** | **n** | **(** | **%** | **)** | **n** | **(** | **%** | **)** | **n** | **(** | **%** | **)** | **n** | **(** | **%** | **)** |
| Age (years) |  |  |  |  |  |  |  |  |  |  |  |  |  |  |  |  |  |  |  |  |  |  |  |  |  |
|  | 18-29 | 608 | ( | 12.2 | ) | 859 | ( | 19.1 | ) | 1646 | ( | 13.5 | ) | 919 | ( | 11.8 | ) | 436 | ( | 8.5 | ) | 628 | ( | 14.8 | ) |
|  | 30-39 | 791 | ( | 15.8 | ) | 860 | ( | 19.1 | ) | 1842 | ( | 15.2 | ) | 1083 | ( | 13.9 | ) | 481 | ( | 9.3 | ) | 593 | ( | 14.0 | ) |
|  | 40-49 | 906 | ( | 18.1 | ) | 948 | ( | 21.1 | ) | 2173 | ( | 17.9 | ) | 1440 | ( | 18.4 | ) | 642 | ( | 12.5 | ) | 647 | ( | 15.2 | ) |
|  | 50-59 | 777 | ( | 15.5 | ) | 720 | ( | 16.0 | ) | 1481 | ( | 12.2 | ) | 1031 | ( | 13.2 | ) | 529 | ( | 10.3 | ) | 477 | ( | 11.2 | ) |
|  | 60-69 | 669 | ( | 13.4 | ) | 504 | ( | 11.2 | ) | 1027 | ( | 8.5 | ) | 763 | ( | 9.8 | ) | 514 | ( | 10.0 | ) | 411 | ( | 9.7 | ) |
|  | 70-79 | 659 | ( | 13.2 | ) | 382 | ( | 8.5 | ) | 1313 | ( | 10.8 | ) | 1173 | ( | 15.0 | ) | 818 | ( | 15.9 | ) | 627 | ( | 14.8 | ) |
|  | 80-89 | 485 | ( | 9.7 | ) | 198 | ( | 4.4 | ) | 2011 | ( | 16.6 | ) | 1190 | ( | 15.2 | ) | 1257 | ( | 24.4 | ) | 721 | ( | 17.0 | ) |
|  | 90-99 | 109 | ( | 2.2 | ) | 24 | ( | 0.5 | ) | 656 | ( | 5.4 | ) | 217 | ( | 2.8 | ) | 476 | ( | 9.2 | ) | 144 | ( | 3.4 | ) |
|  | missing | 0 | ( | 0.0 | ) | 0 | ( | 0.0 | ) | 0 | ( | 0.0 | ) | 0 | ( | 0.0 | ) | 0 | ( | 0.0 | ) | 0 | ( | 0.0 | ) |
|  | *total (n)* | *5004* | *(* | *100.0* | *)* | *4495* | *(* | *100.0* | *)* | *12149* | *(* | *100.0* | *)* | *7816* | *(* | *100.0* | *)* | *5153* | *(* | *100.0* | *)* | *4248* | *(* | *100.0* | *)* |
| Towsend |  |  |  |  |  |  |  |  |  |  |  |  |  |  |  |  |  |  |  |  |  |  |  |  |  |
|  | Least deprived | 913 | ( | 18.2 | ) | 674 | ( | 15.0 | ) | 2067 | ( | 17.0 | ) | 1388 | ( | 17.8 | ) | 966 | ( | 18.7 | ) | 692 | ( | 16.3 | ) |
|  | 2 | 970 | ( | 19.4 | ) | 725 | ( | 16.1 | ) | 2101 | ( | 17.3 | ) | 1394 | ( | 17.8 | ) | 936 | ( | 18.2 | ) | 746 | ( | 17.6 | ) |
|  | 3 | 1032 | ( | 20.6 | ) | 888 | ( | 19.8 | ) | 2797 | ( | 23.0 | ) | 1618 | ( | 20.7 | ) | 1166 | ( | 22.6 | ) | 907 | ( | 21.4 | ) |
|  | 4 | 1117 | ( | 22.3 | ) | 1070 | ( | 23.8 | ) | 2799 | ( | 23.0 | ) | 1709 | ( | 21.9 | ) | 1119 | ( | 21.7 | ) | 971 | ( | 22.9 | ) |
|  | Most deprived | 972 | ( | 19.4 | ) | 1138 | ( | 25.3 | ) | 2385 | ( | 19.6 | ) | 1707 | ( | 21.8 | ) | 966 | ( | 18.7 | ) | 932 | ( | 21.9 | ) |
|  | missing | 0 | ( | 0.0 | ) | 0 | ( | 0.0 | ) | 0 | ( | 0.0 | ) | 0 | ( | 0.0 | ) | 0 | ( | 0.0 | ) | 0 | ( | 0.0 | ) |
|  | *total (n)* | *5004* | *(* | *100.0* | *)* | *4495* | *(* | *100.0* | *)* | *12149* | *(* | *100.0* | *)* | *7816* | *(* | *100.0* | *)* | *5153* | *(* | *100.0* | *)* | *4248* | *(* | *100.0* | *)* |
| Smoking Status |  |  |  |  |  |  |  |  |  |  |  |  |  |  |  |  |  |  |  |  |  |  |  |  |  |
|  | ex-smoking | 845 | ( | 16.9 | ) | 855 | ( | 19.0 | ) | 2339 | ( | 19.3 | ) | 2135 | ( | 27.3 | ) | 1057 | ( | 20.5 | ) | 1133 | ( | 26.7 | ) |
|  | non-smoking | 2437 | ( | 48.7 | ) | 1427 | ( | 31.7 | ) | 5634 | ( | 46.4 | ) | 2738 | ( | 35.0 | ) | 2838 | ( | 55.1 | ) | 1609 | ( | 37.9 | ) |
|  | smoking | 1131 | ( | 22.6 | ) | 1527 | ( | 34.0 | ) | 2790 | ( | 23.0 | ) | 1970 | ( | 25.2 | ) | 786 | ( | 15.3 | ) | 1016 | ( | 23.9 | ) |
|  | missing | 591 | ( | 11.8 | ) | 686 | ( | 15.3 | ) | 1386 | ( | 11.4 | ) | 973 | ( | 12.4 | ) | 472 | ( | 9.2 | ) | 490 | ( | 11.5 | ) |
|  | *total (n)* | *5004* | *(* | *100.0* | *)* | *4495* | *(* | *100.0* | *)* | *12149* | *(* | *100.0* | *)* | *7816* | *(* | *100.0* | *)* | *5153* | *(* | *100.0* | *)* | *4248* | *(* | *100.0* | *)* |
| Drinking Status |  |  |  |  |  |  |  |  |  |  |  |  |  |  |  |  |  |  |  |  |  |  |  |  |  |
|  | non-drinking | 2492 | ( | 49.8 | ) | 1460 | ( | 32.5 | ) | 5754 | ( | 47.4 | ) | 2788 | ( | 35.7 | ) | 2911 | ( | 56.5 | ) | 1653 | ( | 38.9 | ) |
|  | ex-drinking | 869 | ( | 17.4 | ) | 869 | ( | 19.3 | ) | 2388 | ( | 19.7 | ) | 2200 | ( | 28.1 | ) | 1064 | ( | 20.6 | ) | 1151 | ( | 27.1 | ) |
|  | drinking | 1045 | ( | 20.9 | ) | 1290 | ( | 28.7 | ) | 2496 | ( | 20.5 | ) | 1692 | ( | 21.6 | ) | 780 | ( | 15.1 | ) | 926 | ( | 21.8 | ) |
|  | missing | 598 | ( | 12.0 | ) | 876 | ( | 19.5 | ) | 1511 | ( | 12.4 | ) | 1136 | ( | 14.5 | ) | 398 | ( | 7.7 | ) | 518 | ( | 12.2 | ) |
|  | *total (n)* | *5004* | *(* | *100.0* | *)* | *4495* | *(* | *100.0* | *)* | *12149* | *(* | *100.0* | *)* | *7816* | *(* | *100.0* | *)* | *5153* | *(* | *100.0* | *)* | *4248* | *(* | *100.0* | *)* |
| Diabetes Diagnostic | |  |  |  |  |  |  |  |  |  |  |  |  |  |  |  |  |  |  |  |  |  |  |  |  |
|  | no | 4357 | ( | 87.1 | ) | 3986 | ( | 88.7 | ) | 10276 | ( | 84.6 | ) | 6367 | ( | 81.5 | ) | 4147 | ( | 80.5 | ) | 3446 | ( | 81.1 | ) |
|  | yes | 647 | ( | 12.9 | ) | 509 | ( | 11.3 | ) | 1873 | ( | 15.4 | ) | 1449 | ( | 18.5 | ) | 1006 | ( | 19.5 | ) | 802 | ( | 18.9 | ) |
|  | missing | 0 | ( | 0.0 | ) | 0 | ( | 0.0 | ) | 0 | ( | 0.0 | ) | 0 | ( | 0.0 | ) | 0 | ( | 0.0 | ) | 0 | ( | 0.0 | ) |
|  | *total (n)* | *5004* | *(* | *100.0* | *)* | *4495* | *(* | *100.0* | *)* | *12149* | *(* | *100.0* | *)* | *7816* | *(* | *100.0* | *)* | *5153* | *(* | *100.0* | *)* | *4248* | *(* | *100.0* | *)* |
| Height (m) |  |  |  |  |  |  |  |  |  |  |  |  |  |  |  |  |  |  |  |  |  |  |  |  |  |
|  | mean (sd) | 1.6 | ( | 0.1 | ) | 1.8 | ( | 0.1 | ) | 1.6 | ( | 0.1 | ) | 1.7 | ( | 0.1 | ) | 1.6 | ( | 0.1 | ) | 1.7 | ( | 0.1 | ) |
|  | missing | 1786 | ( | 35.7 | ) | 1481 | ( | 32.9 | ) | 4665 | ( | 38.4 | ) | 2832 | ( | 36.2 | ) | 2021 | ( | 39.2 | ) | 1439 | ( | 33.9 | ) |
|  | *total (n)* | *5004* | *(* | *100.0* | *)* | *4495* | *(* | *100.0* | *)* | *12149* | *(* | *100.0* | *)* | *7816* | *(* | *100.0* | *)* | *5153* | *(* | *100.0* | *)* | *4248* | *(* | *100.0* | *)* |
| SBP (mmHg) |  |  |  |  |  |  |  |  |  |  |  |  |  |  |  |  |  |  |  |  |  |  |  |  |  |
|  | mean (sd) | 129.2 | ( | 16.2 | ) | 130.7 | ( | 14.6 | ) | 129.5 | ( | 14.7 | ) | 131.2 | ( | 13.3 | ) | 131.3 | ( | 15.8 | ) | 131.3 | ( | 14.4 | ) |
|  | missing | 1659 | ( | 33.2 | ) | 2099 | ( | 46.7 | ) | 3601 | ( | 29.6 | ) | 2631 | ( | 33.7 | ) | 1280 | ( | 24.8 | ) | 1484 | ( | 34.9 | ) |
|  | *total (n)* | *5004* | *(* | *100.0* | *)* | *4495* | *(* | *100.0* | *)* | *12149* | *(* | *100.0* | *)* | *7816* | *(* | *100.0* | *)* | *5153* | *(* | *100.0* | *)* | *4248* | *(* | *100.0* | *)* |
| LDL-Cholesterol (mmol/L) | |  |  |  |  |  |  |  |  |  |  |  |  |  |  |  |  |  |  |  |  |  |  |  |  |
|  | mean (sd) | 3.3 | ( | 1.1 | ) | 3.3 | ( | 1.1 | ) | 3.3 | ( | 1.1 | ) | 3.1 | ( | 1.1 | ) | 3.3 | ( | 1.1 | ) | 3.1 | ( | 1.1 | ) |
|  | missing | 1901 | ( | 38.0 | ) | 1757 | ( | 39.1 | ) | 4783 | ( | 39.4 | ) | 2797 | ( | 35.8 | ) | 1792 | ( | 34.8 | ) | 1544 | ( | 36.3 | ) |
|  | *total (n)* | *5004* | *(* | *100.0* | *)* | *4495* | *(* | *100.0* | *)* | *12149* | *(* | *100.0* | *)* | *7816* | *(* | *100.0* | *)* | *5153* | *(* | *100.0* | *)* | *4248* | *(* | *100.0* | *)* |
| HDL-Cholesterol (mmol/L) | |  |  |  |  |  |  |  |  |  |  |  |  |  |  |  |  |  |  |  |  |  |  |  |  |
|  | mean (sd) | 1.6 | ( | 0.5 | ) | 1.3 | ( | 0.4 | ) | 1.5 | ( | 0.5 | ) | 1.3 | ( | 0.5 | ) | 1.5 | ( | 0.6 | ) | 1.3 | ( | 0.5 | ) |
|  | missing | 1459 | ( | 29.2 | ) | 1385 | ( | 30.8 | ) | 3596 | ( | 29.6 | ) | 1995 | ( | 25.5 | ) | 1307 | ( | 25.4 | ) | 1089 | ( | 25.6 | ) |
|  | *total (n)* | *5004* | *(* | *100.0* | *)* | *4495* | *(* | *100.0* | *)* | *12149* | *(* | *100.0* | *)* | *7816* | *(* | *100.0* | *)* | *5153* | *(* | *100.0* | *)* | *4248* | *(* | *100.0* | *)* |
| First Dose (mg) |  |  |  |  |  |  |  |  |  |  |  |  |  |  |  |  |  |  |  |  |  |  |  |  |  |
|  | mean (sd) | 6.2 | ( | 4.6 | ) | 7.5 | ( | 4.9 | ) | 84.9 | ( | 111.9 | ) | 95.3 | ( | 129.0 | ) | 1.3 | ( | 1.1 | ) | 1.6 | ( | 1.4 | ) |
|  | missing | 1369 | ( | 27.4 | ) | 1138 | ( | 25.3 | ) | 4865 | ( | 40.0 | ) | 3164 | ( | 40.5 | ) | 1735 | ( | 33.7 | ) | 1396 | ( | 32.9 | ) |
|  | *total (n)* | *5004* | *(* | *100.0* | *)* | *4495* | *(* | *100.0* | *)* | *12149* | *(* | *100.0* | *)* | *7816* | *(* | *100.0* | *)* | *5153* | *(* | *100.0* | *)* | *4248* | *(* | *100.0* | *)* |
| Body Weight (kg) * | |  |  |  |  |  |  |  |  |  |  |  |  |  |  |  |  |  |  |  |  |  |  |  |  |
|  | mean (sd) | 69.7 | ( | 17.2 | ) | 81.1 | ( | 17.5 | ) | 73.3 | ( | 18.3 | ) | 82.5 | ( | 18.0 | ) | 70.1 | ( | 18.2 | ) | 81.6 | ( | 18.7 | ) |
|  | missing | 2156 | ( | 43.1 | ) | 1858 | ( | 41.3 | ) | 5540 | ( | 45.6 | ) | 3438 | ( | 44.0 | ) | 2389 | ( | 46.4 | ) | 1771 | ( | 41.7 | ) |
|  | *total (n)* | *5004* | *(* | *100.0* | *)* | *4495* | *(* | *100.0* | *)* | *12149* | *(* | *100.0* | *)* | *7816* | *(* | *100.0* | *)* | *5153* | *(* | *100.0* | *)* | *4248* | *(* | *100.0* | *)* |
| *(*) Body weight has been calculated as the average of the wieght records available up to 12 months before treatment initiation.* | | | | | | | | | | | | | | | | | | | | | | | | | |

Figure S1. Visual comparison off linear splines models with restricted cubic models of changes in body weight over time before and after treatment initiation, by drug and sex.
